# Clinical assessment and molecular mechanism of the upregulation of Toll-like receptor 2 (TLR2) in myocardial infarction

**DOI:** 10.1186/s12872-022-02754-y

**Published:** 2022-07-15

**Authors:** Ming-Jie Li, Shi-Bai Yan, Hao Dong, Zhi-Guang Huang, Dong-Ming Li, Yu-lu Tang, Yan-Fang Pan, Zhen Yang, Hong-Bo Pan, Gang Chen

**Affiliations:** 1 Department of Pathology/Forensic Medicine, The First Affiliated Hospital of Guangxi Medical University, No. 6 Shuangyong Road, Nanning, 530021 Guangxi Zhuang Autonomous Region People’s Republic of China; 2 Department of Cardiovascular Medicine, The First Affiliated Hospital of Guangxi Medical University, No. 6 Shuangyong Road, Nanning, 530021 Guangxi Zhuang Autonomous Region People’s Republic of China; 3 Department of Pathology, Hospital of Guangxi Liugang Medical Co., LTD./Guangxi Liuzhou Dingshun Forensic Expert Institute, No. 9, Queershan Rd, Liuzhou, 545002 Guangxi Zhuang Autonomous Region People’s Republic of China; 4 Department of Gerontology, NO. 923 Hospital of Chinese People’s Liberation Army, No. 1 Tangcheng Rd, Nanning, 530021 Guangxi Zhuang Autonomous Region People’s Republic of China

**Keywords:** Biomarker, Myocardial infarction, Single-cell RNA-seq, Standardized mean difference (SMD), Toll-like receptor 2 (TLR2)

## Abstract

**Objective:**

The prevalence and mortality of cardiovascular diseases remain ranked first worldwide. Myocardial infarction (MI) is the central cause of death from cardiovascular diseases, seriously endangering human health. The clinical implication of toll-like receptor 2 (TLR2) remains contradictory, and its mechanism is still unknown. Hence, the objective of this study was to elucidate the clinical value and molecular mechanism of TLR2 in MI.

**Methods:**

All high-throughput datasets and eligible literature were screened, and the expression levels of TLR2 were collected from the MI. The integrated expression level of TLR2 was displayed by calculating the standardized mean difference (SMD) and the area under the curve (AUC) of the summary receiver operating characteristic curve (sROC). The related TLR2 genes were sent for pathway analyses by gene ontology (GO), Kyoto encyclopedia of genes and genome (KEGG), and disease ontology (DO). Single-cell RNA-seq was applied to ascertain the molecular mechanism of TLR2 in MI.

**Results:**

Nine microarrays and four reported data were available to calculate the comprehensive expression level of TLR2 in MI, including 325 cases of MI and 306 cases of controls. The SMD was 2.55 (95% CI = 1.35–3.75), and the AUC was 0.76 (95% CI = 0.72–0.79), indicating the upregulation of TLR2 in MI. The related TLR2 genes were primarily enriched in the pathways of atherosclerosis, arteriosclerotic cardiovascular disease, and arteriosclerosis, suggesting the clinical role of TLR2 in the progression of MI. Afterward, TLR2 was upregulated in myeloid cells in MI.

**Conclusions:**

TLR2 may have a crucial role in progressing from coronary atherosclerosis to MI. The upregulation of TLR2 may have a favorable screening value for MI.

**Supplementary Information:**

The online version contains supplementary material available at 10.1186/s12872-022-02754-y.

## Introduction

Myocardial infarction (MI) is mainly caused by coronary atherosclerotic heart disease[1]. The current incidence of MI has remained stubbornly high worldwide, seriously endangering human health in forensic medicine. In recent years, research on MI has gradually deepened from the general histopathology and cellular levels to the molecular level. However, the detailed mechanism of MI is unclear. Thus, molecular MI machinery has constantly been a hot and challenging topic in forensic and clinical medicine research fields [2–4]. Cell signal transduction and gene dysfunctions have been documented to have a substantial role in regulating cell function and disease progress related to MI. However, the existing interpretation of the regulation mechanism of these molecules remains unreliable and unclear [5]. Therefore, it is necessary to explore the specific roles and regulatory mechanisms of those genes contributing to the incidence of MI to ascertain effective methods for early MI diagnosis and treatment.

The Toll-like receptor (TLR) family is crucial in the first line of host defense against microbial infections and innate and adaptive immunity [6]. A diversity of TLRs has a significant position in various signal transduction mechanisms, mainly involved in heart function, body temperature regulation, and energy modulation. Among the 10 TLRs determined in humans, two (TLR2 and TLR4) occur in heart tissues [7]. TLR2 has recently been revealed in recent publications to be linked with the incidence and progress of coronary heart disease. For example, it has been confirmed in several studies that a higher expression of TLR2 may affect the MI process, and the underexpression of TLR2 may have a protective effect on the heart [7–9]. However, all the existing reports have some limitations: the experiments were performed with only a small number of cases from a single research center or only considered a single detecting method [10, 11]. Therefore, the expression and significance of TLR2 in MI remain controversial, requiring further studies. Additionally, a molecular explanation for TLR2 expression has not been provided in most previous reports. Alternatively, the molecular mechanism of TLR2 in MI was limited to a single downstream gene of TLR2 or a single regulating axis [8]. Therefore, we planned this study to illuminate the expression implications of TLR2 in MI and the potential molecular pathway and regulation mechanism.

The deterioration of cardiac function after MI is the result of multi-cell participation, involving not only poor cardiomyocyte regeneration after MI but also the complex role of non-cardiomyocytes, such as endothelial cells (EC), fibroblasts (FB), and macrophages [12, 13]. Therefore, preventing and treating the deterioration of cardiac function after MI is a key and difficult point to determine. Single-cell transcriptome sequencing (scRNA-seq) is a revolutionary ultra-high flux technology for analyzing gene expression profiles at a single-cell resolution. Through unbiased proof of the identity of cell lineages and new cell types, its composition and dynamic change process are analyzed to determine the heterogeneity and plasticity of cells in the whole complex biological system [14]. The scRNA-seq technology has been used to explore the cellular MI mechanism [15]. It is known that there are differences in the transcriptional characteristics of distinct types of cells and cells in diverse pathological regions or at various time points between MI [16, 17]. Therefore, with the application of scRNA-seq, the relationship between the deterioration of cardiac function after MI and various cell lineages can be revealed. Applying this procedure will allow a thorough understanding of the cellular and molecular mechanisms of the deterioration of cardiac function after MI, providing evidence for the search for therapeutic targets.

Hence, all high-throughput MI data and those detected by other methods were collected worldwide. The clinicopathological implications and potential forensic molecular mechanisms of TLR2 in MI were then investigated, with the expectation of assisting in early MI screening and treatment.

## Methods and materials

### Data screening of TLR2 expression levels in MI

There were two parts for collecting the expression levels of TLR2 in MI, including high-throughput methods from public datasets and other detecting strategies from published literature. First, we searched for relevant microarray and RNA-seq data of MI samples from the Gene Expression Omnibus (GEO), Sequence Read Archive (SRA), and ArrayExpress databases. The searching strategy was “myocardial infarction.” The inclusion criteria for the microarray and RNA-seq data were as follows: (1) The species was *Homo sapiens*; (2) The studies should include MI and control groups; (3) The sample size should not be smaller than three; (4) The TLR2 expression levels could be extracted or calculated based on the raw high-throughput data. The exclusion criteria were: (1) The species was not *Homo sapiens*; (2) The data did not contain the non-MI controls; (3) The sample size was smaller than three; (4) The data was duplicated. The raw microarray and RNA-seq data were downloaded, normalized, and log2 transformed.

We also extracted the relevant data from published literature besides the high-throughput data to have a complete overview of the TLR2 expression levels in MI. We searched for eligible publications from several datasets, such as PubMed, Web of Science, Wiley Online Library, Cochrane Google Scholar, EMBASE, Chinese CNKI, Wan Fang, Ovid, LILACS, Chong Qing VIP, Science Direct, Cochrane Central Register of Controlled Trials, Google Scholar, EMBASE, and China Biology Medicine disc. The searching keywords were “myocardial infarction” and “TLR2”.

**Fig. 1:**
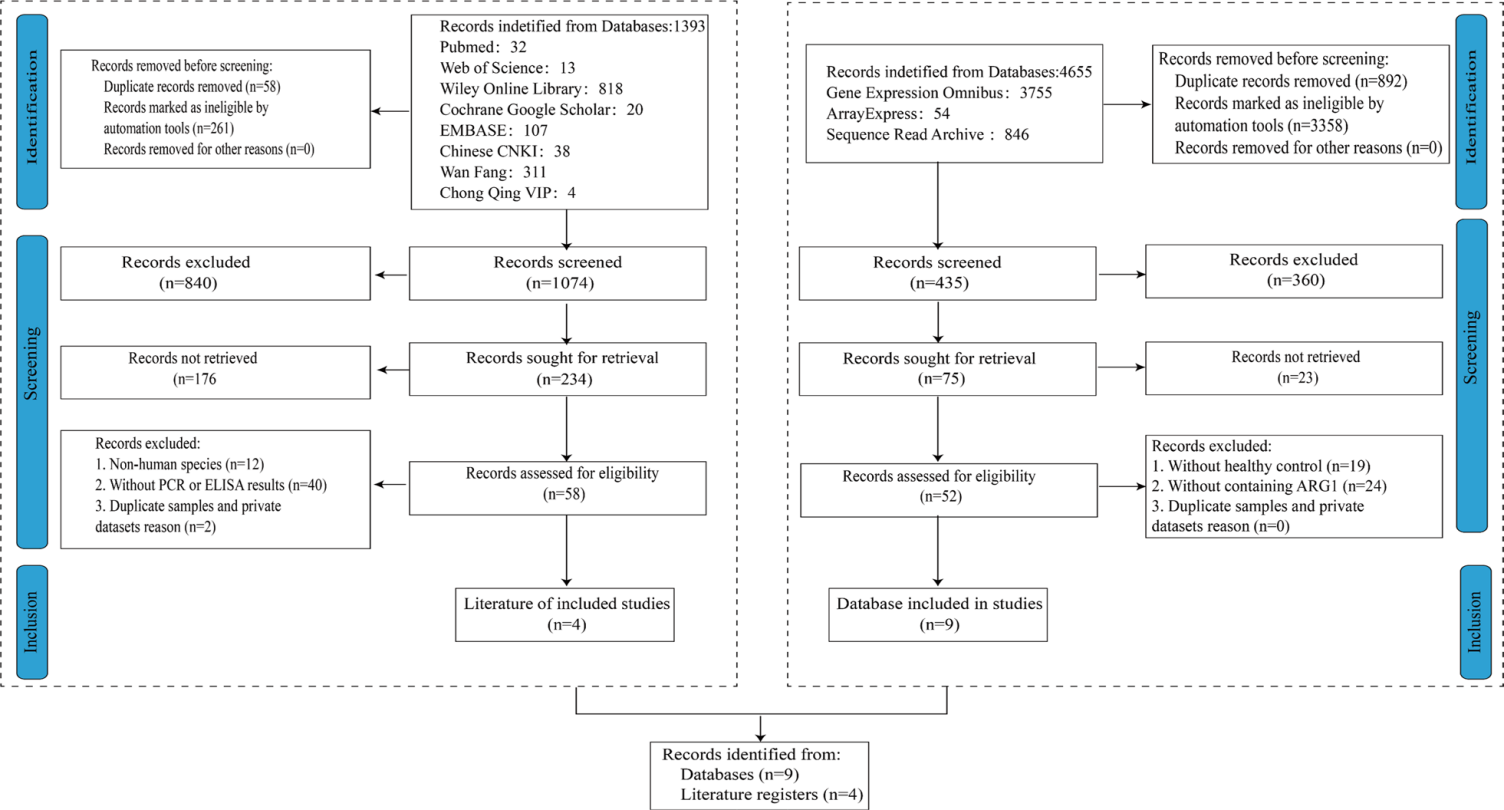
The screening process of all eligible studies of the expression levels of Toll-Like Receptor 2 (TLR2) in myocardial infarction (MI)

The following criteria were required for the included studies: (1) Patients were diagnosed with MI according to the corresponding guidelines; (2) The researches should be case–control studies with at least three cases in each group; (3) TLR2 expression levels between MI and control groups should be evaluated; (4) TLR2 expression in serum or tissues was detected with polymerase chain reaction (PCR) or enzyme-linked immunosorbent assay (ELISA); (5) The reports were published in English or Chinese. Meanwhile, reviews, letters, abstracts of conference, and duplicated studies were excluded (Fig. 1).

Two independent authors (Ming-Jie Li and Hao Dong) checked the full paper of all relevant studies, extracting essential parameters. A third reviewer (Gang Chen) resolved any disagreement between the authors. We extracted the TLR2 expression value in MI and control groups detected with different methods. After the homogeneity of variance was performed, a Student’s *t*-test was conducted to evaluate the TLR2 expression difference between MI and control groups if their variance were equal. Otherwise, a non-parametric test would be conducted. The receiver operating characteristic (ROC) curves were drawn using R, and the area under curves (AUCs) were also obtained.

### Comprehensive analysis of TLR2 expression levels in MI

After TLR2 expression was extracted from each dataset or study, the standardized mean difference (SMD) was calculated by constructing a forest map [18–21]. The I^2^ statistics was used to assess the heterogeneity of the involved data as previously reported [22, 23]. A summary receiver operating characteristic curve (sROC) was generated to check the prospective diagnostic value of TLR2 for MI. Simultaneously, the sensitivity, specificity, positive likelihood ratio, negative likelihood ratio, and odds ratio were also prepared for the expression levels of TLR2 in MI.

### Potential molecular mechanism of TLR2 overexpression in MI

The SMDs of all genes in the above high-throughput datasets were calculated. Genes with SMD > 1 were regarded as upregulated genes in MI and vice versa. The correlation indexes (*r*) were also calculated using Pearson’s coefficient method for all the genes in the high-throughput datasets. The screening conditions for related genes were *P* < 0.05 and |*r*|> 0.5. The intersection of positively-related genes (*r* > 0.5) and highly-expressed genes was performed. Similarly, the negatively related genes (*r* <  − 0.5) intersected with lowly expressed genes. These two groups of genes were used for subsequent pathway analyses performed using the clusterProfiler package (version 4.0.5) [24] of the R language. Gene ontology (GO), Kyoto encyclopedia of genes and genomes (KEGG) [25–27], disease ontology (DO), and protein–protein interaction (PPI) were considered to investigate possible gene annotations, signaling pathways, and protein interactions.

Since TLR8 was obtained according to the correlation degree value to be the most significant related gene of TLR2, the correlations between TLR2 and TLR8 expression levels in all datasets were analyzed with Pearson’s coefficient. The SMDs of TLR8 expression and the relationships between TLR2 and TLR8 were constructed. A P-value < 0.05 was considered statistically significant for all the statistics in the current study.

### scRNA-seq analysis of TLR2 in infarcted myocardium tissue

The GSE145154 dataset of GEO contains six infarcted myocardium tissues for dissecting the transcriptional traits of the cells. The subpopulations of cells were obtained through linear dimensionality reduction principal component analysis (PCA) and uniform manifold approximation and projection (UMAP) clustering. The marker genes and disease difference molecules in the cell subpopulations were analyzed and screened using the Wilcox rank-sum test. The marker genes of each population obtained in this dataset were compared with the marker molecules collected in the study, providing raw data and CellMarker. According to the relative expression of the single-cell genes after integrating and correcting the batch effect, the differentially expressed gene set was selected as the variable to conduct the trajectory construction function. Then, a single-cell development trajectory diagram was built in the Monocle 2 software package, which was displayed with 2D visual results.

CellphoneDB (Release v2.1.7) [28] is a database containing ligands, receptors, and their interactions. The communication molecules between cells and the study of the communication networks between different cell types can be comprehensively and systematically analyzed with CellphoneDB. The proportion of cells expressing the gene and the mean gene expression value were calculated for the gene expressed by the cell population. The possibility of cell-type specificity for a specified receptor-ligand complex is expressed by calculating the proportion of the average equal to or higher than the actual average. If the observed mean is in the first 5%, the P-value of the interaction is < 0.05.

Protein activity influence in single cells (PISCES) [29] is a method based on a regulatory network for analyzing single-cell gene expression profiles. Vastly variable and noisy single-cell gene expression profiles were transformed with PISCES into robust and repeatable protein activity profiles. Generally, the activity of up to 6,000 regulatory proteins from single-cell gene expression profiles can be precisely evaluated by PISCES to substantially improve the capability to examine the biological function and correlation of gene products.

### Statistical analysis

In this study, alongside the statistical analysis explained above, when TLR2 expression had a normal distribution, R 4.0.3 was used to conduct a Student’s *t*-test. If TLR2 expression was abnormally distributed, a non-parametric test was used to compare the differential expression of TLR2 in the experimental and control groups. The AUC was used to assess the capacity of TLR2 expression to discriminate between the experimental and control groups. STATA 14.0 was employed to calculate the SMD and 95% confidence interval, systematically reflecting the expression of TLR2. The sROC was calculated to demonstrate the capacity of TLR2 to distinguish the experimental group from the control group.

## Results

### Screening procedure for TLR2 expression levels in MI

By the comprehensive search of different nine throughput databases and relevant literature, 12 independent studies were eventually included in our research, including nine microarray datasets (GSE19339, GSE29532, GSE34198, GSE48060, GSE60993, GSE66360, GSE97320, GSE109048, and GSE141512) and another four publications [30–33] (Fig. 1). Finally, all the high-throughput data included were microarrays and no RNA-seq. Each dataset was strictly under the corresponding MI diagnosis guidelines. This rigorous experimental design was approved by the relevant review committee. In general, these studies were based on the following three characteristics: abnormal myocardial biomarkers, typical symptoms (thoracodynia), typical ECG (pathological Q wave), ST-segment elevation, or depression (Table S2, Table S3). All the included control members were healthy people, excluding chronic diseases mainly containing cardiovascular disease, diabetes, and tumor.

### Significant overexpression levels of TLR2 in MI tissues

**Fig. 2:**
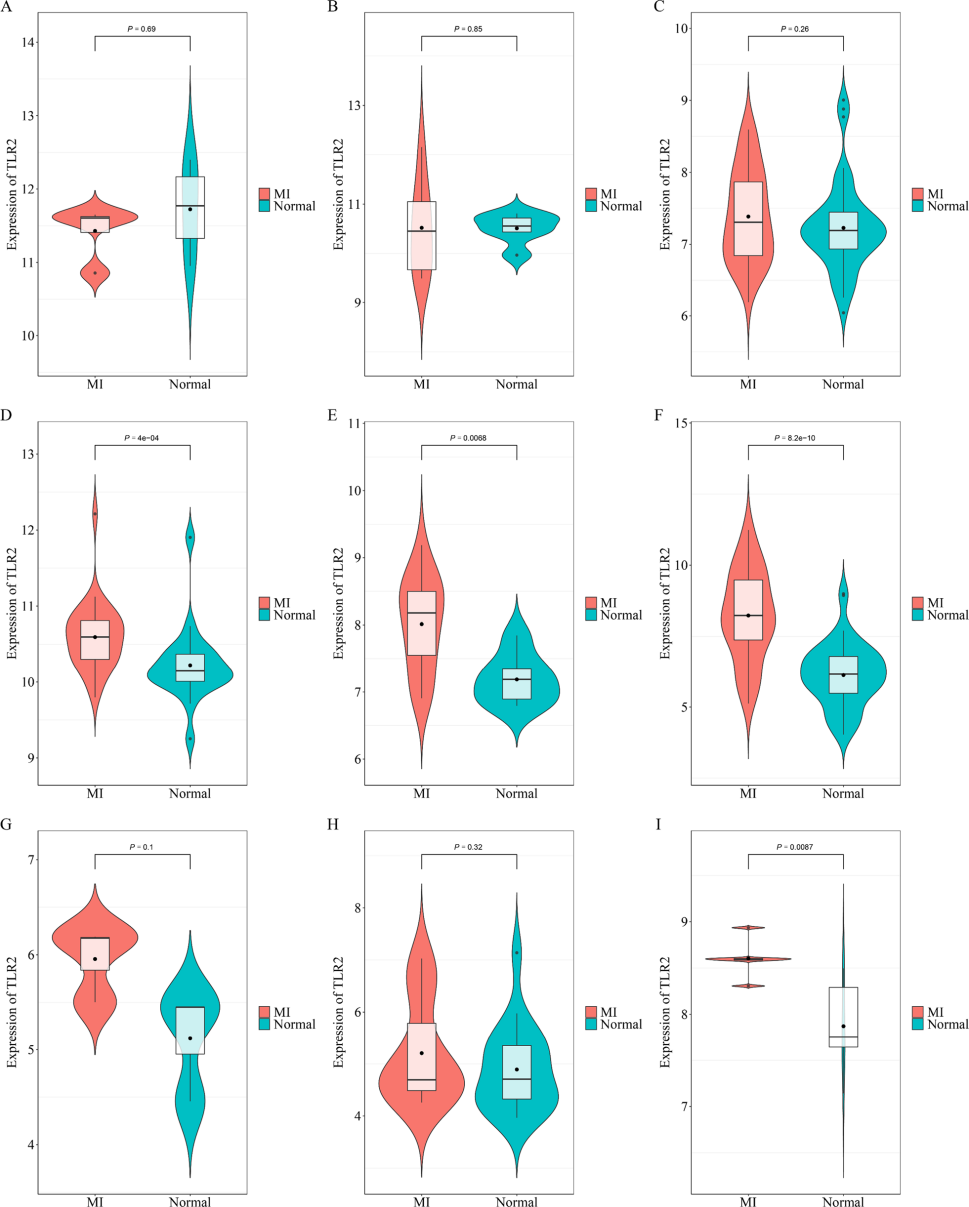
The expression levels of TLR2 between the MI and control group from high throughput datasets. **A** GSE19339, **B** GSE29532, **C** GSE34198, **D** GSE48060, **E** GSE60993, **F** GSE66360, **G** GSE97320, **H** GSE109048, **I** GSE141512

TLR2 expression levels of the MI group were markedly increased than that in the non-MI control groups (*P* < 0.05) in four microarrays (GSE48060, GSE60993, GSE66360, GSE141512, Fig. 2).

Because of the inconsistency between all involved studies, it was essential to perform an integrated analysis to show the overall trend of TLR2 expression in MI by combining all available data. The overall SMD was 2.55, combining all nine microarrays and four publications with 325 cases of MI and 306 cases of non-MI controls. Since I^2^ was equal to 96.8% and potential heterogeneity was indicated by the *P* < 0.05, there was an apparent heterogeneity with the fixed model, supporting the use of the random-effects model. The microarray GSE34198 was suggested by the sensitivity assessment as a potential heterogeneity source. It was indicated by Begg’s test (*P* = 0.127) and Egger’s test (*P* = 0.06) that there was no apparent publication bias.

**Fig. 3:**
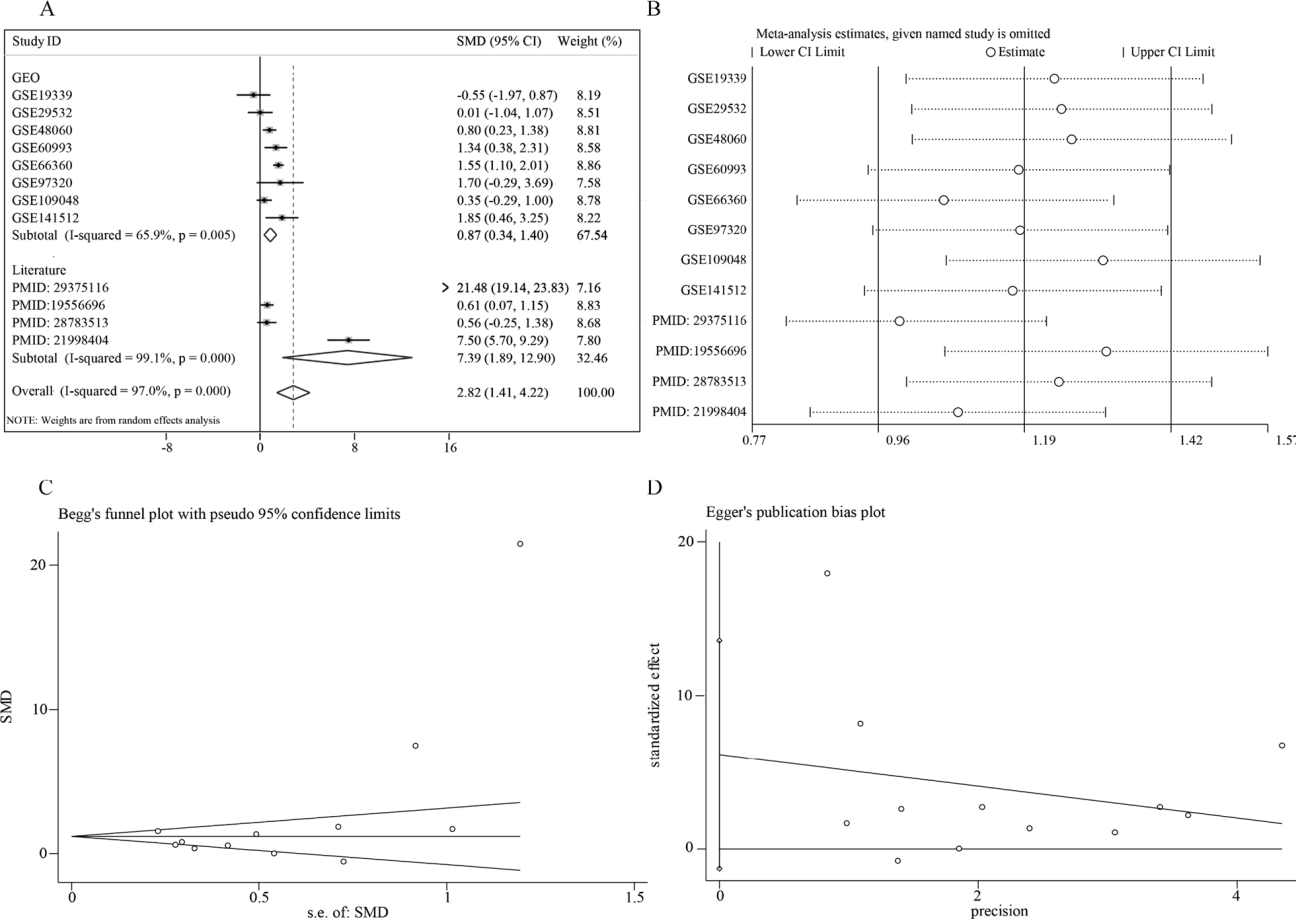
Comprehensive TLR2 expression levels of MI after the omission of GSE34198. **A** Forest plot of TLR2 expression, **B** sensitivity analysis, **C** Begg's test, D. Egger's test

A subgroup analysis was performed after omitting the data from GSE34198 to achieve a more precise TLR2 expression level in MI. It was shown with this procedure that different detecting methods yielded distinct SMDs, even though both were higher than zero. An SMD of 0.87 was generated by nine microarrays, while it was much higher, equal to 7.39, in the four other publications. Using the random-effects model, the overall SMD was 2.82, with 325–45 cases of MI and 306–48 cases of non-MI controls (Fig. 3).

**Fig. 4:**
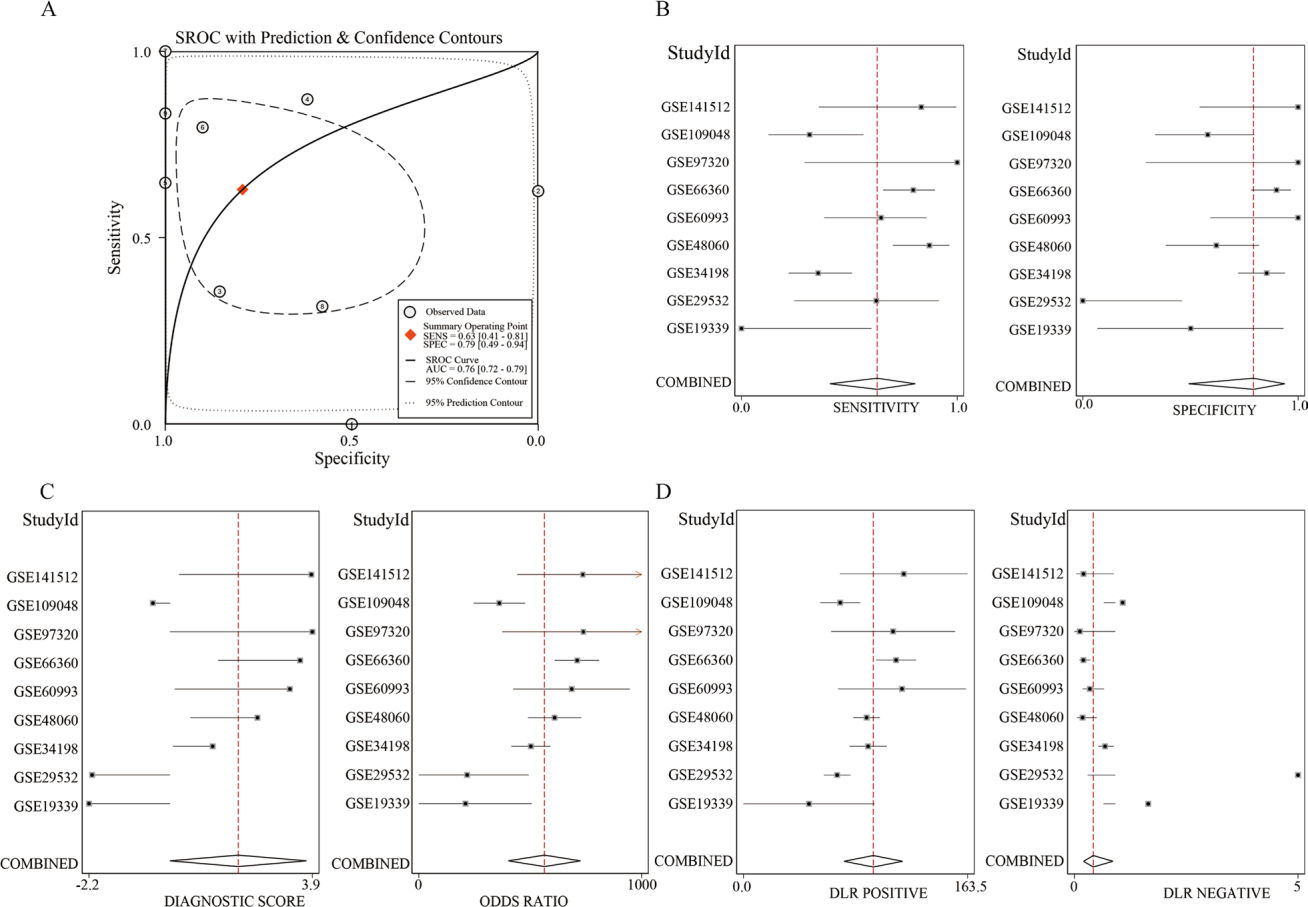
Potential differentiating ability of TLR2 overexpression in MI. **A** sROC curve, **B** Sensitivity and specificity, **C** Positive and Negative likelihood ratio, **D** Odds ratio

Another statistical method concerning ROC was utilized to verify the different TLR2 expression levels in MI. ROCs were drawn for all nine microarrays, and the AUCs fluctuated in a relatively large range. The sROC was constructed to obtain a more comprehensive and objective statistical result. Therefore, all datasets were combined, and the final AUC was 0.76 for TLR2 upregulation to identify MI from the non-MI control groups (Fig. 4A). Meanwhile, the specificity was 0.79 (95% CI 0.49–0.94), and the sensitivity was 0.63 (95% CI: 0.41–0.81) (Fig. 4B–D). This added to evidence that TLR2 overexpression could have a fundamental part in the progression of MI.

### Probable molecular mechanism of the overexpression level of TLR2 in MI

Since the differentially expressed genes (DEGs) have an essential role in MI, we obtained 1,127 upregulated and 1,065 downregulated genes after the SMD of every gene involved in the nine microarrays were generated. Meanwhile, the co-expressed genes were also calculated, and the number of positively co-expressed genes of TLR2 was 9452, and 9600 for negatively co-expressed ones. The intersection of upregulated and positively co-expressed TLR2 genes yielded 950 genes. At the same time, 934 genes were obtained at the intersection of downregulated and negatively co-expressed genes. After PPI was performed, 49 and 55 hub genes were shown from the two groups mentioned above for subsequent analyses.

It was displayed in the GO and KEGG analyses of the upregulated and positively co-expressed genes that the gene annotation of “Neutrophil Activation” was ranked the highest in the biological process (BP). The “secretory granule membrane” was the top one in cellular components (CC) analysis. The “pattern recognition receptor activity” apparated to the top one in the molecular function (MF) analysis. For KEGG, the first potential signaling pathway was “osteoclast differentiation”. For DO, most interestingly, the first three pathway enrichments were “atherosclerosis,” “arteriosclerotic cardiovascular disease,” and “arteriosclerosis,” all of which are well-documented diseases closely related to MI. The first pathway of “atherosclerosis” from the DO analysis contained 50 genes.

Meanwhile, it was shown in the GO and KEGG analyses of the downregulated and negatively co-expressed genes that the first gene annotation in BP was “ribonucleoprotein complex biogenesis”. In CC, these genes were primarily enriched in the “mitochondrial matrix”. In MF, the involved genes largely participate in “ATPase Activity”. More importantly, it was uncovered with KEGG that these genes were chiefly related to the pathways of the “cell cycle”. The first pathway was “primary immunodeficiency Disease” in the DO analysis.

Among all the positively co-expressed genes, TLR8 had the highest degree with TLR2 in PPI. Based on eight independent microarrays, we then showed the relationships between TLR8 and TLR2, which had TLR2 and TLR8 expression data. An integrative co-correlation index of 0.84 (95% CI: 0.80–0.88) was also identified.

### TLR2 is suggested by scRNA-seq analysis to be upregulated on myeloid cells in infarcted myocardium tissue

**Fig. 5:**
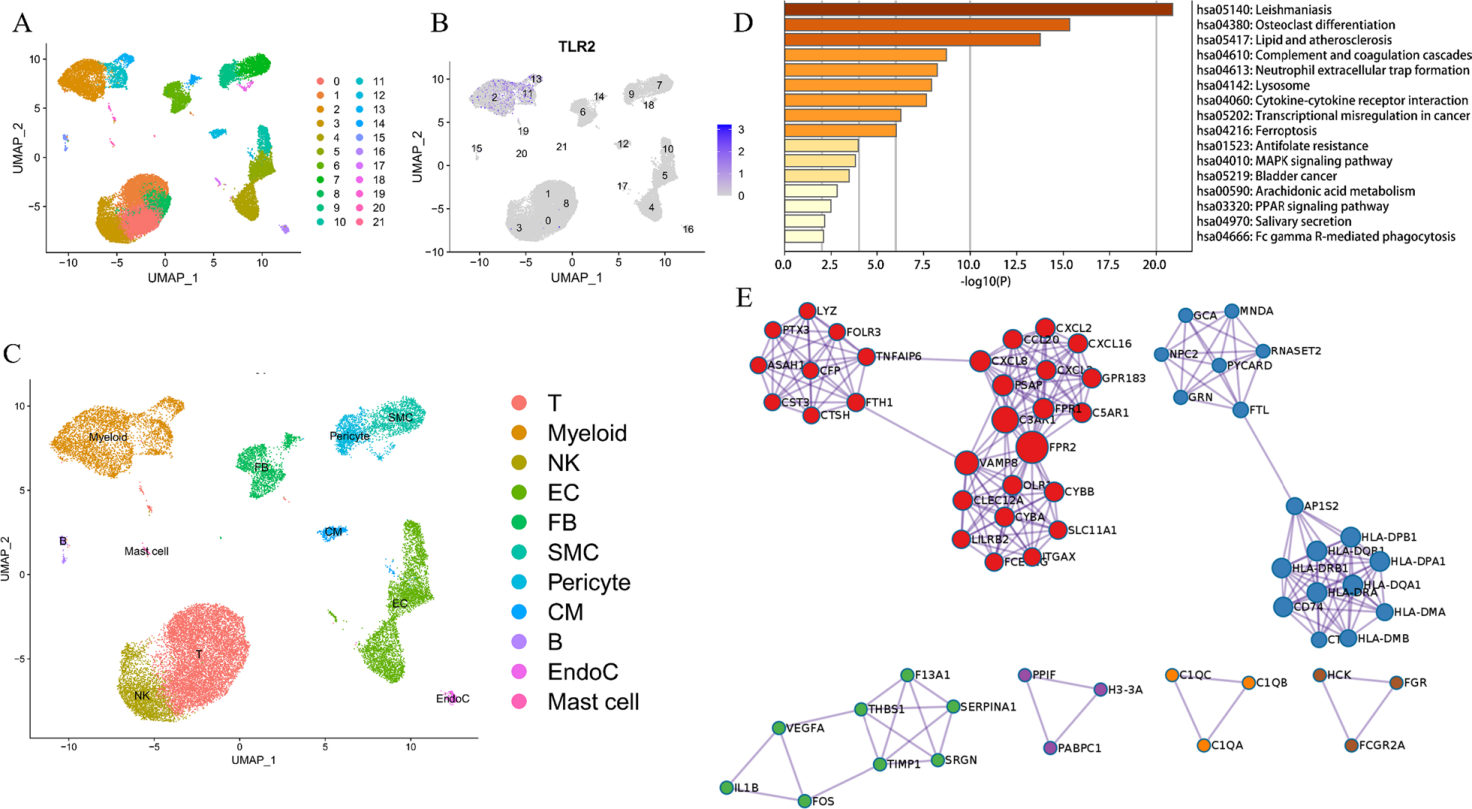
scRNA-seq analysis process of GSE145154. **A** Uniform manifold approximation and projection (UMAP) distribution of 22 annotated cell types in MI tissues. **B** Distribution of TLR2 expression in cell clusters of MI tissues. **C** UMAP distribution of 11 annotated cell types in MI tissues. **D** KEGG pathway [25–27] analysis of Myeloid cells in MI tissues. **E** Mcode module of upregulated genes on Myeloid cells in MI tissues.

**Fig. 6:**
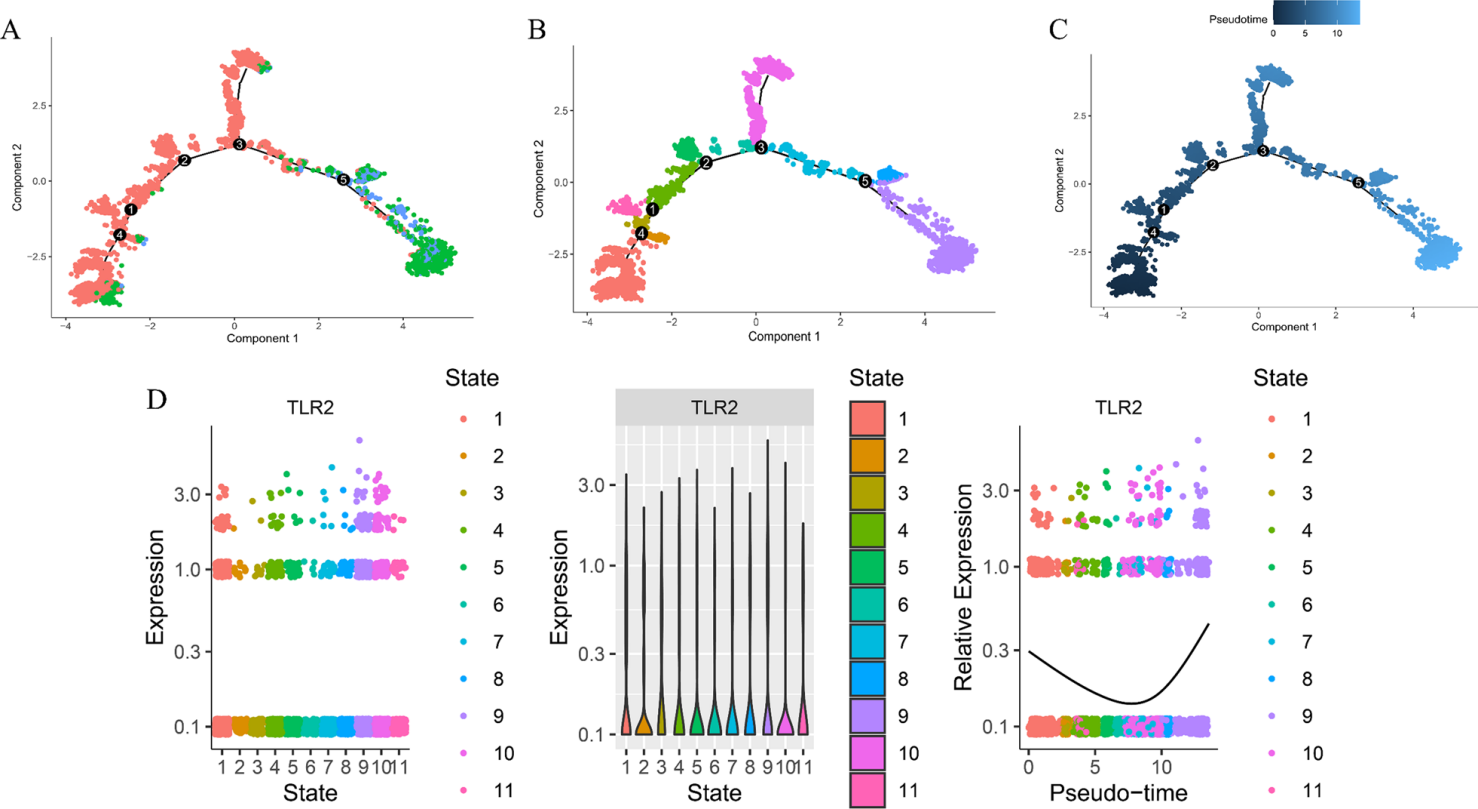
Trajectory analysis of Myeloid cells in MI tissue. **A** The two-dimensional trajectory distribution map of cluster annotated on Myeloid cells. **B** The two-dimensional distribution map of state shows the trajectory results of Myeloid cells. **C** Display diagram of cell development timing score calculated according to development trajectory analysis. **D** The dynamic changes of the expression level of TLR2 during development

**Fig. 7:**
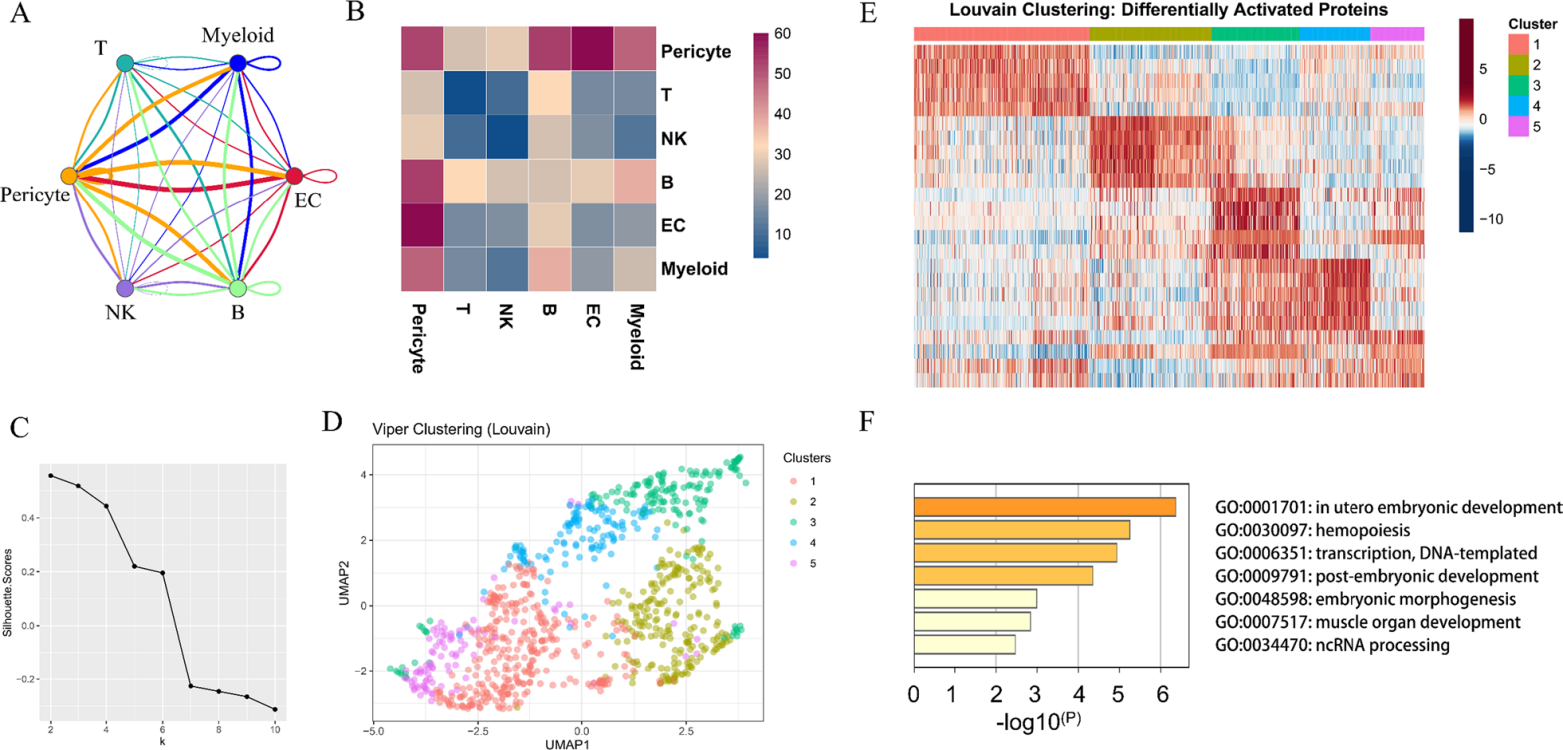
CellPhoneDB (Release v2.1.7) [28] and PISCES analysis of GSE145154. **A** Network diagram of cells expressing TLR2. **B** Heatmap of interaction level of 6 kinds of cells expressing TLR2. **C** K-nearest neighbor (KNN) graph by evaluating viper similarity distance. **D** Uniform manifold approximation and projection (UMAP) distribution of Viper clustering. **E** Proteins inferring differentially expressed on 5 clusters. **F** GO function enrichment analysis of differentially expressed proteins

The harmony function was adopted to rectify the sample heterogeneity of the expression matrix. The cells were identified as different clusters using an unsupervised clustering method. The upregulated molecules of the cell subpopulation were identified through differential screening (Fig. 5A). Markers collected in the CellMarker database and marker molecules collected in the paper providing raw data were compared with upregulated genes. Overall, the cell subpopulation was marked as cells (Fig. 5C) in infarcted myocardium tissue [T cell, myeloid cells, NK cell, endothelial cells (ECs), fibroblasts (FBs), smooth muscle cells (SMCs), pericyte, cardiomyocyte (CM), B cells, endocardial cells (EndoC), mast cell]. TLR2 was significantly overexpressed in myeloid cells (Fig. 5B). Genes specifically upregulated myeloid cells enriched in KEGG pathways[25–27] including neutrophil extracellular trap formation, complement and coagulation cascades, lipid, and atherosclerosis biological processes (Fig. 5D–E). Differentially expressed genes among the cell groups were taken as the reference gene set. Then, a pseudo-temporal algorithm was employed to predict the dynamic development trajectory of cell subpopulation (Fig. 6A–C) and TLR2 expression level (Fig. 6D). Simultaneously, it was found by cell communication analysis that the development of MI was promoted by the interaction between myeloid cells and pericyte promoted (Fig. 7A–B). Then, the ARACNE algorithm was used to infer transcriptome interactions from gene expression data. The expression of transcription factors was then used in the Viper algorithm to deduce the activity of the protein regulatory networks. After calculating the protein activity, PAM clustering was performed on the distance matrix based on the protein activity. By evaluating the similarity, the number of clusters was determined to be 2 (Fig. 7C). A single-cell gene expression profile can be transformed into a meta-cell profile by PISCES to promote the quality of the ARACNE network. The differentially expressed proteins displayed by the differential protein analysis of the five subpopulations (Fig. 7D) were primarily concentrated in biological pathways, such as organ development and hematopoietic function (Fig. 7E–F).

## Discussion

The most vital point of the current study is that the comprehensive upregulation of TLR2 mRNA is supported by different detection methods combining clinical cases from multiple independent cohorts from 11 countries. Furthermore, the DEGs from all the possible high-throughput datasets were calculated and further compromised with the related TLR2 genes in MI. It was demonstrated in the signaling pathway analyses that TLR2 could be involved in some pre-diseases of MI. The prospective mechanism by which TLR2 overexpression causes MI was partially revealed in this process.

One of the highlights of our study is analyzing the available nine microarrays and four studies from multiple countries and races, using SMD, ROC, SROC, KEGG, and DO statistics and data analysis methods to explore TLR2 expression in 325 MI patients. Our results are more convincing when using various test methods than only a single test. Additionally, we proved that TLR2 co-expressed genes are enriched in the gene group of atherosclerosis and atherosclerotic cardiovascular disease, further suggesting that TLR2 may play a vital part in the progress of coronary atherosclerosis to MI.

The upregulated expression of TLR2 in MI has been reported in previous studies. For example, in the study by Zhang et al., the mRNA expression of TLR2 in PBMC of MI patients was found to be significantly higher than the control by using RT-PCR with MI (n = 84) and healthy controls (n = 82) [30]. Selejan et al. also reported that TLR2 was overexpressed based on 20 cases with flow cytometry (Ref 28, PMID 21998404). Additionally, Hally et al. found that platelet-TLR2 was insignificantly upregulated in AMI patients (Ref 27, PMID 28783513). It was mentioned in the studies above that only a single detecting method was used, and the experimental subjects were recruited from one institute. These experimental settings may be responsible for some errors and bias in their conclusions. We collected and used an integrated method to analyze extensive data related to TLR2 expression levels. We examined a total of 325 patients with MI and 306 healthy controls in nine microarrays and four studies to provide a comprehensive overview of TLR2 in MI. Therefore, we believe that our research results are more convincing. It was shown in our expression study that in the four microarrays (GSE48060, GSE60993, GSE66360, and GSE141512), the TLR2 expression level in the MI group was markedly increased compared to that in the non-MI groups. At the same time, the other five microarrays had no significant difference. Among the five insignificant datasets, the upward trend of TLR2 in MI patients was reflected in the three datasets. A more sufficient sample size may make the upward TLR2 regulation more significant. It was shown in two other datasets that TLR2 was downregulated in MI patients. However, these two datasets had only four and eight experimental group samples, which may cause some errors and bias. Currently, the urgent need for early detection, early diagnosis, and timely treatment of MI for the relatively low sensitivity and specificity cannot be met by existing serum markers (cTn, MB, and CK-MB) [34]. It was revealed in the results of this study that the area under the ROC curve was over 0.5, indicating that TLR2 expression has a particular diagnostic value for MI, and the total AUC of the sROC curve was 0.78. These results were indications that a distinction between diseased and non-pathological tissues could be detected with TLR2 expression, potentially becoming a new MI diagnostic marker.

In this study, it was shown with DO analysis that TLR2-positive-related genes were mainly enriched in arteriosclerosis, arteriosclerotic cardiovascular disease, and atherosclerosis, indicating that TLR2 may be involved in the development of atherosclerosis into MI. It was revealed in recent publications that TLR2 has a pivotal role in inflammation, contributing to the occurrence and development of AS [35–37]. TLR2 can participate in AS development by combining with MyD88 to activate the NF-κB signaling pathway [38]. Schoneveld et al. used the apolipoprotein E (ApoE)-/- atherosclerosis mouse model to activate TLR2 to increase the formation of AS plaques, indicating that TLR2 can promote AS [39]. AS causes plaque rupture and thrombosis to contribute to MI, which is also one of the leading causes of cardiovascular death [40, 41]. TLR2, related to both AS and MI, can be used to assess the risk of AS and MI with a particular screening value.

More importantly, TLR2 was found to be overexpressed specifically in myeloid cells by scRNA-seq analysis. Myeloid cells include neutrophils, monocytes, macrophages, and dendritic cells [42]. Neutrophils can produce reactive oxygen species (ROS) with significant cytotoxicity [43]. With the ROS and matrix degradation protease released by neutrophils, endothelial cell dysfunction may be aggravated, and blood vessels may be damaged, resulting in the occurrence and development of atherosclerosis [44]. The rupture of unstable atherosclerotic plaque is the leading cause of AMI [45]. In the process of MI, damps will be released into the blood from the necrotic myocardium, activating the systemic immune system, inducing the differentiation and recruitment of monocyte macrophages, even to aggravate ischemic MI [46]. Macrophages are an essential part of the injury response. After MI, different macrophages have diverse roles, such as M1 macrophages mediating proinflammatory response and M2 macrophages presenting anti-inflammatory response [13, 47]. Concurrently, myocardial healing after MI mainly depends on scar repair, including the inflammatory, proliferative, and maturity stages [48]. After MI, innate immunity is activated rapidly to produce a significant and transient adaptive inflammatory response [49]. The monocytes/macrophages in the blood may express the M1 phenotype and with many proinflammatory cytokines, such as tumor necrosis factor α (TNF- α), Interleukin-1 (IL-1), IL12, IL-23, interferon-γ, and ROS being secreted. Ischemic injury may be aggravated by such proinflammatory cytokines, contributing to pathophysiological processes, such as cardiac remodeling and heart failure [50]. At the same time, the monocytes/macrophages can express the M2 phenotype, removing damaged necrotic cells and matrix fragments, having an adaptive anti-inflammatory response, and participating in the repair of the injured myocardium [51]. It was indicated in the results above that TLR2 may be involved in the activation of M1 macrophages during the MI pathological process.

However, certain limitations remain in our research. First, because some included datasets lack corresponding clinical information, this study may not exclude the potential impact of some factors except MI on TLR2 expression. Second, this study lacks experimental verification, with which the alteration in the expression of TLR2 between the MI group and the control group, such as WB and IHC, can be evaluated. The reason for this is that clinical-pathological specimens drawn from living organisms, such as cancer and tumors, cannot be used in this study to conduct experiments. Second, with the chip selected, the study of most of the myocardial specimen tissue may be allowed after death. The expressed experimental results may deviate from the actual results due to the difference in the time of death. The included sample source contained blood and other body fluid samples as the research object. There may be unstable indicators that need to be repeatedly verified clinically later.

Despite the problems mentioned above, a significant step forward has been provided by this study. The results remain reliable from the summary analysis of larger-scale, multi-center, high-throughput data. It was found in this study found that TLR2 has a potential biomarker or a supplementary indicator of MI or AMI. Nevertheless, extra work is essential to validate the specific mechanism of TLR2 in myocardial infarction and to clarify its application value role in clinical diagnosis and forensic pathology identification.

## Conclusion

TLR2 may have a crucial role in the progression from coronary atherosclerosis to MI. The upregulation of TLR2 may have a favorable MI screening value.

## Supplementary Information

**Additional file 1: Figure S1.** Comprehensive analysis of TLR2 expression levels of MI. **Figure S2.** The ROCs of the overexpression levels of TLR2. **Figure S3.** Differentially expressed genes of MI from nine microarrays shown by volcano maps. **Figure S4.** The TLR2 coexpressed genes in MI. **Figure S5.** The gene annotation and signaling pathways of TLR2 positively-related genes in MI. **Figure S6.** The gene annotation and signaling pathways of TLR2 negatively-related genes in MI. **Figure S7.** The correlations between TLR2 and TLR8 expression in MI. **Table S1.** Information of the involved data concerning TLR2 expression in MI. **Table S2.** Clinical features of nine included microarrays. **Table S3.** Clinical features of four included literatures.

## Data Availability

The datasets presented in this study [including GSE19339, GSE29532, GSE34198, GSE48060, GSE60693, GSE66360, GSE97320, GSE109048, GSE141512, and published articles (PMID: 29375116, PMID: 19556696, PMID: 28783513, and PMID: 21998404)] can be found in online repositories: Gene Expression Omnibus (GEO, https://www.ncbi.nlm.nih.gov/geo/) and PubMed (https://www.ncbi.nlm.nih.gov/)
