# Supplementary material for: Clinical assessment and molecular mechanism of the upregulation of Toll-like receptor 2 (TLR2) in myocardial infarction
Source: BMC Cardiovasc Disord. 2022 Jul 15;22:314. doi: 10.1186/s12872-022-02754-y (PMC9287878; doi:10.1186/s12872-022-02754-y)
Supplement: Supplementary file 1 — Additional file 1: Figure S1. Comprehensive analysis of TLR2 expression levels of MI. Figure S2. The ROCs of the overexpression levels of TLR2. Figure S3. Differentially expressed genes of MI from nine microarrays shown by volcano maps. Figure S4. The TLR2 coexpressed genes in MI. Figure S5. The gene annotation and signaling pathways of TLR2 positively-related genes in MI. Figure S6. The gene annotation and signaling pathways of TLR2 negatively-related genes in MI. Figure S7. The correlations between TLR2 and TLR8 expression in MI. Table S1. Information of the involved data concerning TLR2 expression in MI. Table S2. Clinical features of nine included microarrays. Table S3. Clinical features of four included literatures. [file 12872_2022_2754_MOESM1_ESM.docx]

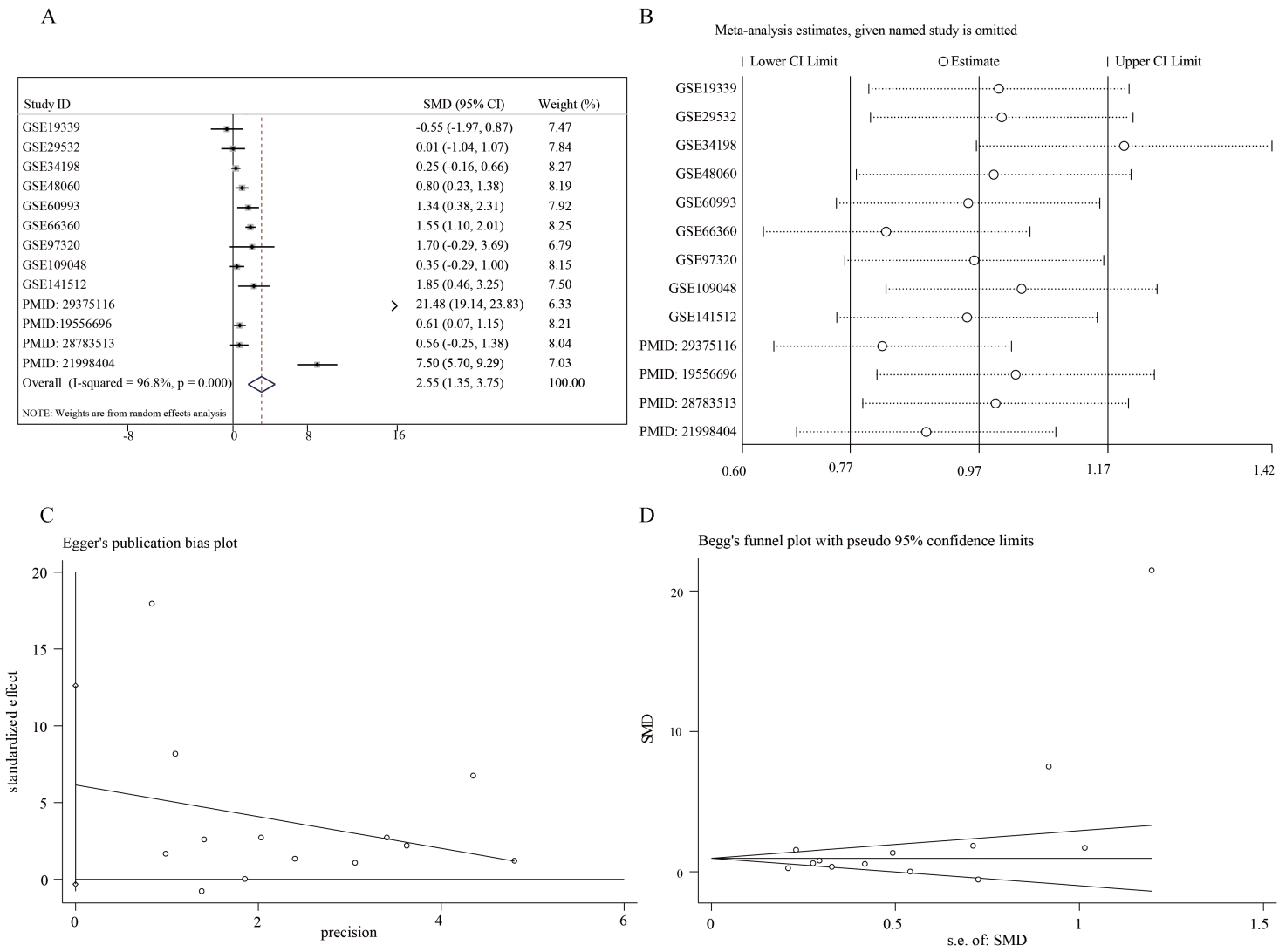


**Figure S1. Comprehensive analysis of TLR2 expression levels of MI**

A: Forest plot of TLR2 expression,

B. Sensitivity analysis,

C. Begg's Test,

D. Egger's test.


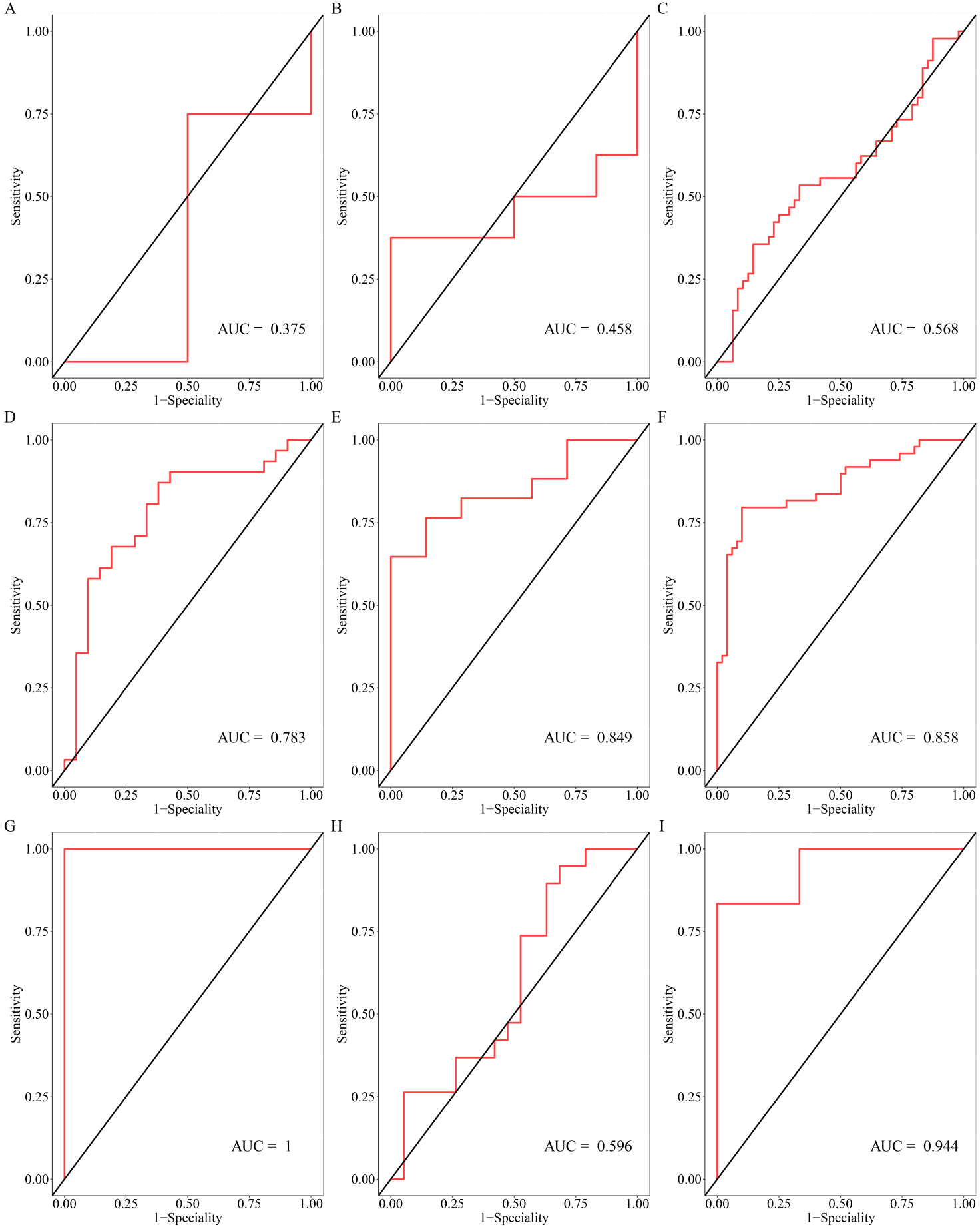


**Figure S2. The ROCs of the overexpression levels of TLR2**

A: GSE19339,

B: GSE29532,

C: GSE34198,

D: GSE48060,

E: GSE60993,

F: GSE66360,

G: GSE97320,

H: GSE109048,

I: GSE141512.


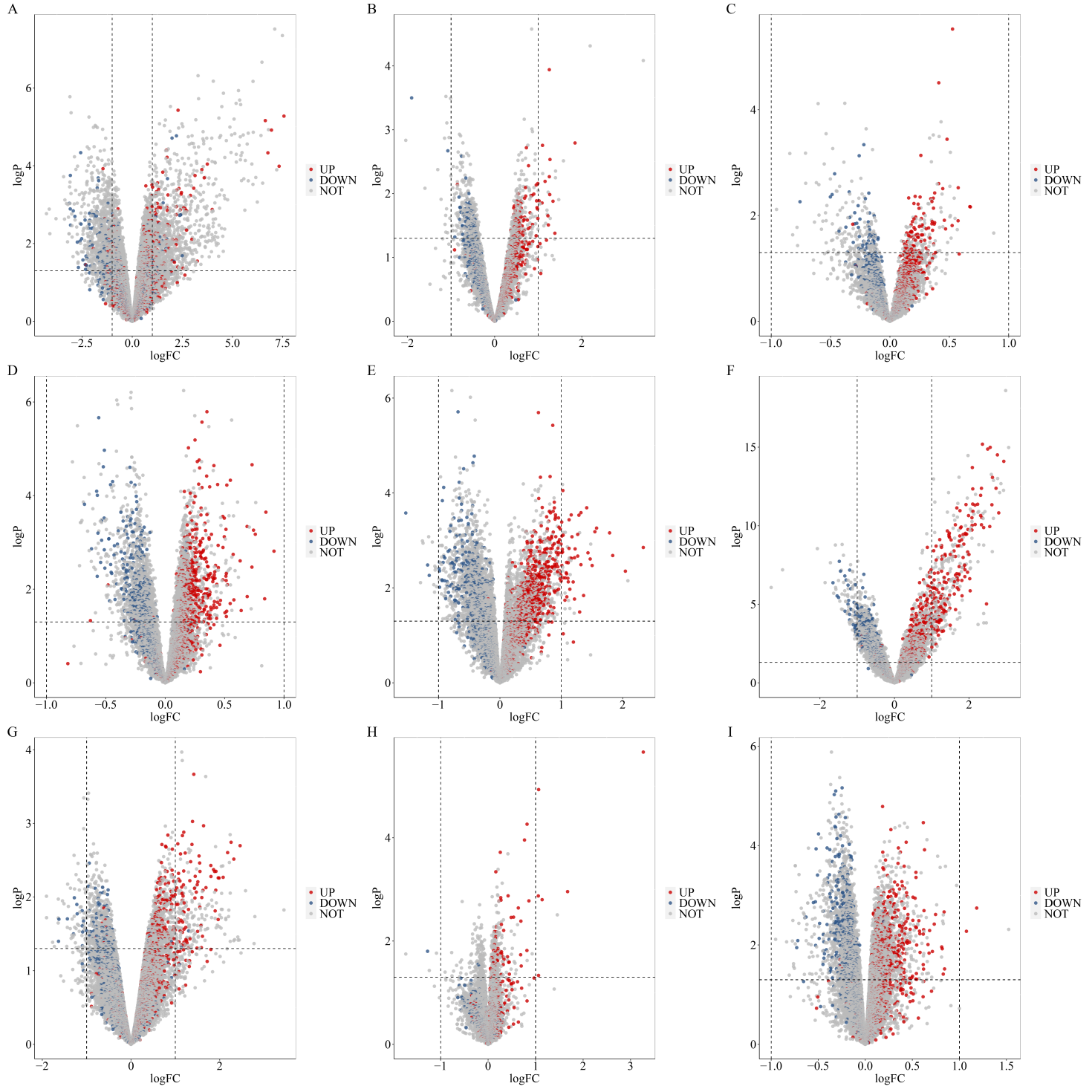


**Figure S3. Differentially expressed genes of MI from nine microarrays shown by volcano maps**

A: GSE19339,

B: GSE29532,

C: GSE34198,

D: GSE48060,

E: GSE60993,

F: GSE66360,

G: GSE97320,

H: GSE109048,

I: GSE141512.

Red dots: upregulated genes; Blue dots: downregulated genes; Gray dots: no obvious changed genes.


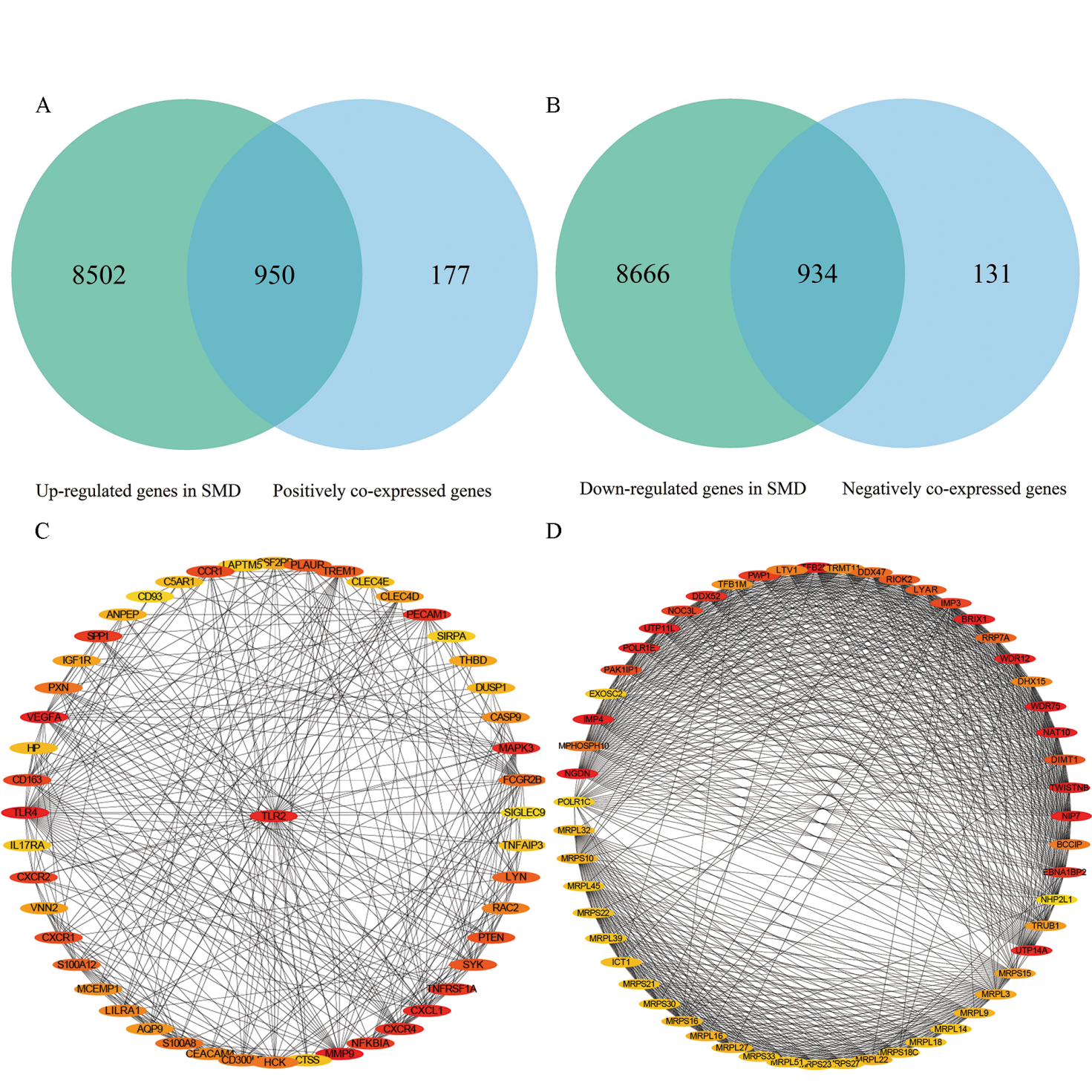


**Figure S4. The TLR2 coexpressed genes in MI**

A: Venn plot between upregulated and positively coexpressed genes,

B: Venn plot between downregulated and negatively coexpressed genes,

C: PPI of genes from Figure 8A,

D: PPI of genes from Figure 8B.

**
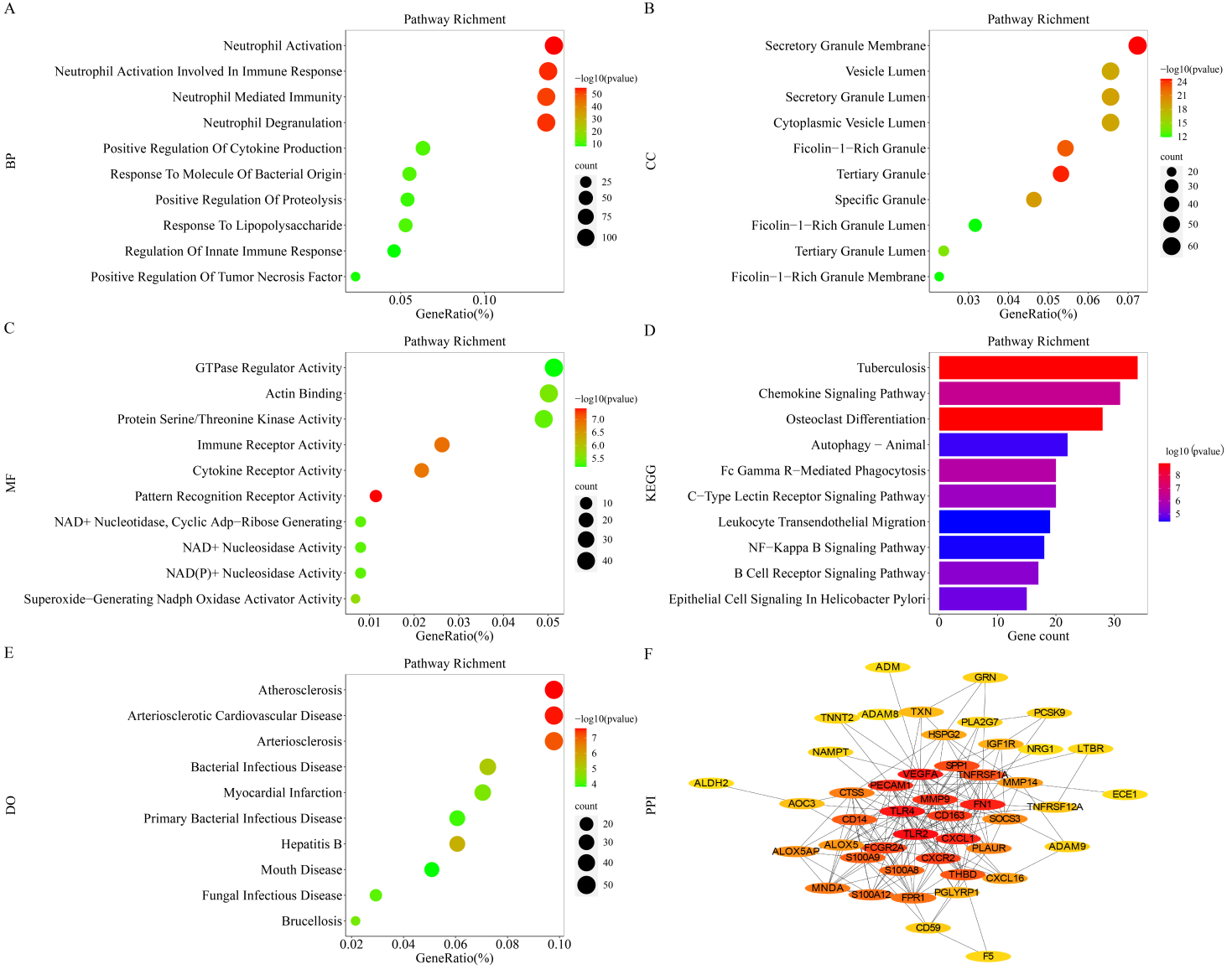
Figure S5. The gene annotation and signaling pathways of TLR2 positively-related genes in MI**

A. Biological Process (BP),

B. Cellular Component (CC),

C. Molecular Function (MF),

D. Kyoto Encyclopedia of Genes and Genome (KEGG),

E. Disease Ontology (DO);

F. Protein-protein Interaction (PPI)


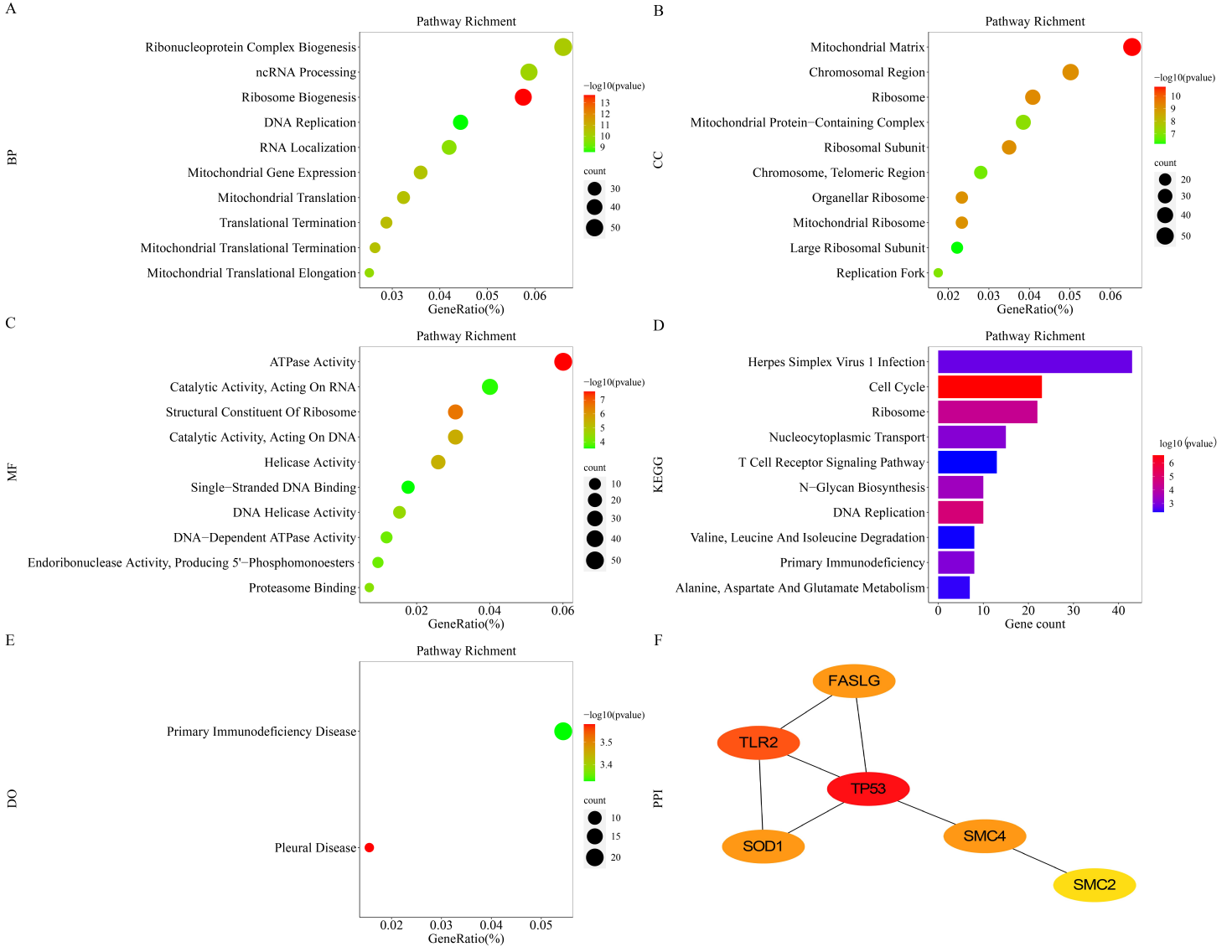


**Figure S6. The gene annotation and signaling pathways of TLR2 negatively-related genes in MI**

A. Biological Process (BP),

B. Cellular Component (CC),

C. Molecular Function (MF),

D. Kyoto Encyclopedia of Genes and Genome (KEGG),

E. Disease Ontology (DO);

F. Protein-protein Interaction (PPI).


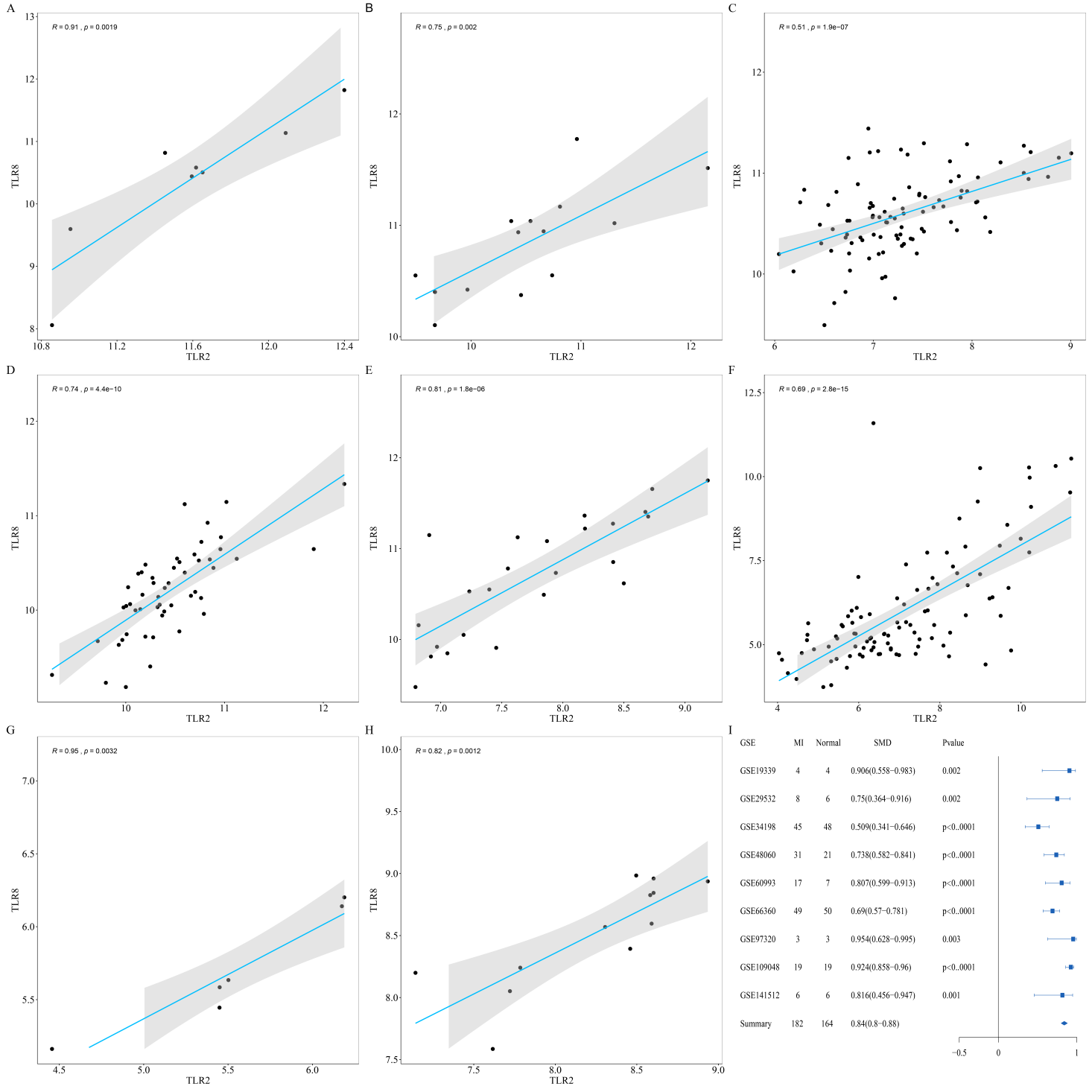


**Figure S7. The correlations between TLR2 and TLR8 expression in MI**

A: GSE19339,

B: GSE29532,

C: GSE34198,

D: GSE48060,

E: GSE60993,

F: GSE66360,

G: GSE97320,

H: GSE141512­­.

I: SMD of the correlation indexes.

**Table S1. Information of the involved data concerning TLR2 expression in MI**

| **ID** | **Region** | **Year** | **Time of data collection** | **N (EXP)** | **Mean (EXP)** | **SD (EXP)** | **N (CTL)** | **Mean (CTL)** | **SD (CTL)** |
| --- | --- | --- | --- | --- | --- | --- | --- | --- | --- |
| GSE19339 | Switzerland | 2011 | Unkonwn | 4 | 11.430 | 0.383 | 4 | 11.724 | 0.647 |
| GSE29532 | Spain | 2013 | The time of admission | 8 | 10.521 | 0.926 | 6 | 10.51 | 0.306 |
| GSE34198 | Czech Republic | 2014 | Unknown | 45 | 7.385 | 0.633 | 48 | 7.228 | 0.613 |
| GSE48060 | USA | 2014 | The time to symptoms onset ranged within 48 hours | 31 | 10.594 | 0.445 | 21 | 10.222 | 0.492 |
| GSE60993 | South Korea | 2015 | The time to symptoms onset within 4 hours | 17 | 8.014 | 0.685 | 7 | 7.186 | 0.375 |
| GSE66360 | USA | 2015 | The time of admission | 49 | 8.226 | 1.571 | 50 | 6.126 | 1.092 |
| GSE97320 | China | 2017 | Unknown | 3 | 5.955 | 0.393 | 3 | 5.119 | 0.574 |
| GSE109048 | Italy | 2019 | The time to symptoms onset ranged from 1 to 6 hours | 19 | 5.206 | 0.955 | 19 | 4.892 | 0.808 |
| GSE141512 | Russia | 2019 | The time to symptoms onset ranged from 24 to 36 hours | 6 | 8.603 | 0.2 | 6 | 7.87 | 0.522 |
| PMID: 29375116 | China | 2018 | The time to symptoms onset within 2 hours | 84 | 2.017 | 0.054 | 82 | 1.012 | 0.038 |
| PMID:19556696 | Japan | 2009 | Before PCI | 27 | 4.8 | 2.8 | 28 | 3.3 | 2.1 |
| PMID: 28783513 | New Zealand | 2017 | Before PCI | 12 | 1.23 | 0.46 | 12 | 1 | 0.35 |
| PMID: 21998404 | Germany | 2012 | After PCI | 20 | 111.1 | 8.2 | 20 | 66.9 | 1.5 |

TLR2: Toll-like receptor 2, MI: Myocardial infarction, n: number of cases involved, SD: The standard deviation, EXP: experimental, CTL: control, PCI: percutaneous coronary intervention.

**Table S2: Clinical features of nine included microarrays.**

| **Study** | **GSE19339** | **GSE29532** | **GSE34198** |
| --- | --- | --- | --- |
| **Patient number** | 4 | 8 | 45 |
| **Gender (male %)** | NP | 8 (100%) | 31 (69%) |
| **Average age** | NP | 53.00 | 63.13 |
| **Time of drawing materials** | NP | NP | NP |
| **Tissue** | Peripheral blood | Peripheral blood | Peripheral blood |
| **Cell type** | Leukocytes | Peripheral blood cells | Peripheral blood cells |
| **Hypertension (%)** | NP | 7 (88%) | NP |
| **Diabetes mellitus (%)** | NP | 0 | 13 (29%) |
| **Treatment history** | None | None | ACEI, Beta blocker, Diuretic, Ca blocker, Statin, Fibrate |
| **Study** | **GSE48060** | **GSE60993** | **GSE66360** |
| **Patient number** | 27 | 17 | 49 |
| **Gender (male %)** | 16 (59%) | NP | 39 (80%) |
| **Average age** | 56.59 | NP | 61.50 |
| **Time of drawing materials** | Within 48 hours after symptoms onset | Within 4 hours after symptoms onset | NP |
| **Tissue** | Peripheral blood | Peripheral blood | Peripheral blood |
| **Cell type** | Peripheral blood cells | Peripheral blood cells | CD146+ Circulating Endothelial Cells |
| **Hypertension (%)** | 18 (67%) | NP | NP |
| **Diabetes mellitus (%)** | 3 (11%) | NP | NP |
| **Treatment history** | Statin, Aspirin, ACEI, Beta blocker | NP | NP |
| **Study** | **GSE97320** | **GSE109048** | **GSE141512** |
| **Patient number** | 3 | 19 | 6 |
| **Gender (male %)** | NP | 15 (79%) | 6 (100%) |
| **Average age** | NP | 65.50 | 51.30 |
| **Time of drawing materials** | NP | Within 6 hours after symptoms onset | NP |
| **Tissue** | Peripheral blood | Peripheral blood | Peripheral blood |
| **Cell type** | Peripheral blood cells | Platelet | Peripheral blood cells |
| **Hypertension (%)** | NP | 5 (26%) | 3 (50%) |
| **Diabetes mellitus (%)** | NP | 5 (26%) | 0 |
| **Treatment history** | NP | None | None |

NP: Not provided, ACEI: Angiotensin converting enzyme inhibitor.

**Table S3: Clinical features of four included literatures.**

| **Study** | **PMID:**  **29375116** | | **PMID:**  **19556696** | **PMID:**  **28783513** | **PMID:**  **21998404** |
| --- | --- | --- | --- | --- | --- |
| **Patient number** | 44 | | 27 | 12 | 20 |
| **Gender (male %)** | 22 (50%) | | 6 (22%) | NP | 12 (60%) |
| **Average age** | 53.00 | | 71.00 | NP | 68.00 |
| **Time of drawing materials** | Within 2 hours after symptoms onset | | NP | NP | NP |
| **Tissue** | Peripheral blood | | Peripheral blood | Peripheral blood | Peripheral blood |
| **Cell type** | PBMCs | | PBMCs | Platelet | CD14+ monocytes |
| **Hypertension (%)** | 29 (66%) | 13 (48%) | | NP | 7 (35%) |
| **Diabetes mellitus (%)** | 16 (37%) | NP | | NP | 7 (35%) |
| **Treatment history** | None | Nitroglycerin | | Aspirin, Clopidogrel, Ticagrelor | Interventional therapy |

NP: Not provided.
